# In Vitro and In Vivo Characteristics of Olive Oil as Excipient for Topical Administration

**DOI:** 10.3390/pharmaceutics14122615

**Published:** 2022-11-26

**Authors:** Marta Rodríguez-Torrado, Aytug Kara, Susana Torrado, Alejandro Romero, Antonio Juberías, Juan J. Torrado, Dolores R. Serrano

**Affiliations:** 1 Department of Pharmaceutics and Food Technology, Complutense University of Madrid, 28040 Madrid, Spain; 2 Galenical and Industrial Pharmaceutical Institute, Complutense University of Madrid, 28040 Madrid, Spain; 3 Department of Pharmacology and Toxicology, Complutense University of Madrid, 28040 Madrid, Spain; 4 Centro Militar de Farmacia de la Defensa (CEMILFARDEF), Base Logística de San Pedro, Colmenar Viejo, 28770 Madrid, Spain

**Keywords:** olive oil, wild olive oil, liquid paraffin, rosehip oil, melatonin, stability, antioxidant, dermoanalyser, MVA

## Abstract

Oily excipients are vital components of dermatological products. In this study, the in vitro and in vivo characteristics of Wild Olive Oil (WOO) were compared with two other types of olive oils: Extra Virgin Olive Oil (EVOO) and Virgin Olive Oil (VOO). This work has also included Liquid Paraffin (LP) and Rosehip Oil (RO) as reference oils. Melatonin was used in the study as a model drug to demonstrate the antioxidant capacity of the oils. The melatonin carrier capacity and antioxidant performance was related to the degree of unsaturation of the oils and was highest for RO and WOO and lowest for LP. However, the most stable oil to oxidation was LP. The in vivo performance of the oils in the skin of eight healthy volunteers was investigated with a dermoanalyser. The highest increment of oil and hydration in the skin was obtained with RO. The lowest perception of oiliness was described for WOO, which produced the highest increase in elasticity of the skin area where it was applied. An in vitro-in vivo correlation was therefore performed through multivariable analysis (MVA).

## 1. Introduction

Olive oil is a protective and hydrating excipient used in many traditional and modern dermatological formulations [1]. The topical application of different types of oils have different effects on the skin depending on their chemical composition and the skin characteristics. For example, the natural antioxidant and anti-inflammatory components of olive oil have been associated with photoprotection, anti-aging, dermatitis prevention and improvement in the appearance of scars and striae [2,3,4,5]. In this work, we compared Wild Olive Oil (WOO), obtained from *Olea europaea var. sylvestris*, with conventional olive oils from *Olea europaea var. europaea*. Two other oils, Liquid Paraffin (LP) and Rosehip Oil (RO), were also incorporated into this study as reference products. In comparison to conventional olive oils, WOO has a higher content of volatile and phenolic compounds (including antioxidants), a higher ratio of shorter chain length oil (C16), such as palmitoleic acid, and a lower ratio of longer chain length oil (C18) [6,7,8,9,10].

Differences in the chemical structure and composition of the oil excipients included in dermatological products are essential for its direct potential action on the skin and its interaction with the formulation’s other components. Dermatological marketed products are usually formulated to contain many active substances, frequently with low aqueous solubility and poor stability characteristics. In this work, melatonin has been included as an example of an active cosmetic anti-aging ingredient [11,12,13,14,15]. The hypothesis underpinning this work is that different oil chemical structures and compositions can significantly affect the overall properties of the dermatological formulations in the skin. This correlation has never been verified and can guide the development of future topical formulations. The following in vitro oil characteristics were evaluated: density, viscosity, melatonin P_lipid/water_ coefficient, melatonin degradation, half-life, and oil oxidation under stress conditions. Differences in the melatonin carrier capacity (estimated as melatonin P_lipid/water_ coefficient) and the effect of the oils on their chemical degradation of melatonin were also studied. 

The hydration and elasticity of the skin can be studied using a Corneometer and a Cutometer, respectively. Both types of probes are components of dermoanalysers which are potent devices for analysing the effect of topical treatments on skin properties. Dobrev describes the methodology of a dermoanalyser to evaluate the increase in oil, hydration, and elasticity in the skin of volunteers following oil application [16]. The efficacy of the oil was expressed as changes in the initial hydration and elasticity values after its topical application [17]. Although both parameters tend to be directly related, sometimes just one of them may be altered. For instance, in the work of Dobrev [17], elasticity was modified but not hydration, while Wissing and Müller [18] observed changes in hydration but not in elasticity. Interestingly, most of the studies [19,20,21,22,23] reported increases in both the elasticity and hydration parameters. The increase in hydration is usually approximately double the increase in elasticity.

The main aim of this work was to study the possible correlation between the in vitro physical and chemical characteristics of the different oil excipients with their in vivo effects on the skin of healthy volunteers. The correlation was conducted by multivariable analysis (MVA) [24,25].

## 2. Materials and Methods

The WOO was a gift obtained from trees in the Sierra Mágina nature reserve (Jaén, Spain) (Dominus^®^ acebuche, Monva SL, Jaén, Spain). The other oils studied were LP (Chemir), RO (Guinama), EVOO (Montabes^®^, Monva SL, Jaén, Spain) and VOO Ph. Eur. (Fagrón Ibérica SAU). The melatonin was Ph. Eur. Grade, purchased from Fagrón Ibérica SAU. All other chemicals were of analytical grade and were used without further purification.

### 2.1. In Vitro Evaluation of the Oils

#### 2.1.1. Density (ρ)

The oils were measured at 20 ± 2 °C in a 25 mL volumetric flask using an analytical weight balance (Entris II essential, Sartorious).

#### 2.1.2. Dynamic Viscosity (η)

Dynamic Viscosity was performed by the capillary viscometer method described in Section 2.2.9 in the Eur. Phar. [26]. The capillary viscometer was calibrated to calculate the viscometer constant (*K*) through the following Equation (1):

K = *η/ρ t* (1)

where *η* is the dynamic viscosity, *ρ* is the density of the certified materials, and *t* is the flow time for the certified reference materials.

Kinematic viscosity (*ʋ*) was evaluated by Equation (2):

*ʋ* = K *t* (2)

where *t* is the time required for the liquid to flow through the marks of the capillary viscometer.

The dynamic viscosity (*η*) was calculated by Equation (3):

*η* = K *t ρ* (3)

where K is the viscometer constant, *t* is the time required for the test material to flow through the capillary viscometer and *ρ* is the density of the test substance.

#### 2.1.3. Melatonin Carrier Capacity Evaluated as P_lipid/water_ Coefficient

The melatonin carrier capacity of the different tested oils was evaluated as the lipid/water partition coefficient through the ratio of melatonin concentrations in both liquids. Transparent glass flasks with a 70 mL capacity were used. First, 25 mL of the test oil was poured into the flask, then 10 mg of melatonin was added, and the closed flask was manually shaken. Finally, 12 mL of purified water was added, and the container was shaken again for 1 min. The container was left to rest for 15 min and the samples were taken from the water and oil phases. The samples from the water phase were filtered using a hydrophilic polypropylene 0.45 µm filter (Filter-Lab PP syringe filter, Anoia SA, Barcelona, Spain), and the melatonin concentration was assayed by HPLC. The melatonin in the oil phase was evaluated as follows: 0.5 mL of the oil phase was transferred to a glass test tube and mixed with 4.5 mL of purified water; the tube was closed and shaken for 30 s, then centrifuged for 5 min at 5000 rpm. The samples taken from the water phase were filtered and assayed by HPLC. The transparent 70 mL glass flasks were stored, protected from light, at 20 ± 5 °C. The samples were withdrawn at three day intervals and the melatonin concentration was assayed for up to 12 days.

The melatonin analytical assay was performed by HPLC according to a validated method described in the USP 38 dietetic supplements [27] and using a Jasco HPLC. The stationary phase was a C18 column with a 10 µm internal particle size (Waters Spherisorb^®^ S10 ODS1, 4.6 × 200 mm). The mobile phase was a mixture of 75:25 (buffer: acetonitrile HPLC gradient grade). The buffer was prepared by dissolving 0.5 g of monopotassium phosphate in 1 L of purified water. Orthophosphoric acid was added to adjust the pH to 3.5. The isocratic flow rate was 1.5 mL/min. At this condition, the typical working pressure was approximately 5.8 MPa. The limit of detection was 0.05 µm/mL and the limit of quantification was 0.15 µg/mL. The injection volume was 10 µL and the wavelength used was 222 nm. Retention times for melatonin and its main product of degradation were 7.7 and 5.5 min, respectively.

#### 2.1.4. Melatonin Degradation and Half-Life

The oil’s antioxidant capacity can delay melatonin degradation. To test this effect, the water phase described in Section 2.1.3 was replaced by a H_2_O_2_ solution at 5% (JVF pharmaceutical company, C.N. 184654.9). Melatonin degradation was evaluated on days 1, 5 and 11. Melatonin degradation half-life was estimated by adjusting the degradation rate to a first-order kinetic process.

#### 2.1.5. Initial Peroxide Value (IP) and Oil Oxidation under Stress Conditions

The IP was evaluated at time 0 and after 30 days of contact between the tested oils and the aqueous phase with and without H_2_O_2_ in the 70 mL glass flasks described in Section 2.1.3. Oil degradation was estimated based on the peroxide value (Ip) described in Section 2.5.5 of the Eur. Phar. [26]. Oil oxidation was evaluated as the difference (ΔIp) between the peroxide values obtained in diluted H_2_O_2_ and in purified water and expressed as a percentage.

### 2.2. In Vivo Evaluation of the Oils

The in vivo study was conducted after approval from the ethics committee of the Complutense University of Madrid CE_20210715-01_SAL. A total of eight Caucasian skin color subjects participated in the study after being fully informed of the protocol to follow. Four males and four females with a median age of 56 and 37 years, respectively, participated in the study. They were distributed randomly according to a cross-over design to test the following oils: WOO, LP, RO, EVOO and VOO Ph. Eur. (Table 1).

Before starting the study, all of the subjects washed their hands with a pH 5.5 hand soap. Each oil (100 µL) was poured onto the back of the hand of each subject using a micropipette. Subjects did not wash the back of their hands till the last measurement was obtained at the end of each period. A washing period of seven days was established among periods for each subject. The subjects were aware of the type of oil applied in each period. The back of the hand was selected because this part of the skin is usually dry and not in contact with clothes. A dermoanalyser Multi Dermascope MDS 800^®^ supplied by Microcaya SL (Bilbao, Spain) was used to quantify the initial values of hydration, skin elasticity and oil levels, consecutively, at 0 and after 15, 30, 60, 120, 240 min and 20 h post-application. The results were expressed as the increment (expressed in %) in oil, hydration, and elasticity compared to values at time 0.

The Multi Dermascope is equipped with three different probes. The Corneometer probe gauges the hydration of the skin by measuring the capacitance of a dielectric medium, where any change in the dielectric constant caused by a variation in the hydration of the epidermis alters the capacitance, which translates into a modification in skin hydration. Located on the probe head there is a fine piece of glass to ensure that only the capacitance changes caused by water content are identified. Based on this, small changes in the water content of the stratum corneum can be detected. The depth of measurement is 10–20 µm, ensuring that deeper skin layers do not influence the measurement; the latter takes place over a short period (1 s) to minimise occlusion effects.

The Cutometer probe measures the skin’s elasticity through the suction and relaxation method. The probes generate a negative pressure, and the skin is pulled towards the opening of the probe. The penetration of the skin is measured using an optical system. Inside the probe is a non-contact optical measuring system, consisting of a light source projected across the aperture to a light receiver, measuring the distance into the aperture that the skin travels. The Cutometer assesses the skin’s resistance to suction, also known as firmness, and its ability to return to its original state, known as elasticity.

The third probe is a Sebumeter that measures the fat levels in the skin based on grease spot photometry of 64 mm^2^. The measurement cassette contains a special tape that becomes transparent when it comes into contact with the sebum on the surface of the skin. The cassette is introduced into an aperture on the Multi Dermascope where the transparency of the tape is measured by sending light through the tape, using a light source in the aperture. The light is reflected by a mirror behind the tape and the transparency is measured by a photocell. The cassette is placed on the skin where the oil was applied for a defined length of time (usually 10 s) and then the cassette was returned to the aperture. The change in the amount of light transmission shows the sebum content of the tape [28].

### 2.3. Statistical Data Assay

Statistical tools for the Student’s t and ANOVA tests were performed in Excel (Microsoft Office 365. The multivariable assay was carried out with Unscrambler^®^ X software (CAMO Software, Norway). The following variables were studied: density, viscosity, melatonin carrier capacity (P_lipid/water_), melatonin degradation, oxidation of the oil, oil retention in the skin between its application and after 20 h evaluated as area under the curve (AUC Δoil (% h)), time after application of maximum oil presence in the skin (Tmax Δoil (h)), maximum increment of oil in the skin (Xmax Δoil (%)), oil remnants in the skin 20 h post-application (X20h Δoil (%)), increment of skin hydration between time 0 and 20 h post-application expressed as area under the curve (AUC ΔHydration (% h)), time after application of maximum hydration value in the skin (Tmax ΔHydration (h)), maximum increment of hydration in the skin (Xmax ΔHydration (%)), hydration remnants in the skin 20 h post-application (X20h ΔHydration (%)), increment of elasticity in the skin between application and after 20 h evaluated as area under the curve (AUC ΔElasticity (% h)), time after application of the maximum increase in the elasticity in the skin (Tmax ΔElasticity (h)), maximum increment of elasticity in the skin (Xmax ΔElasticity (%)), increase in elasticity remnants in the skin 20 h after application (X20h ΔElasticity (%)). Principal Component Assay (PCA) and Multiple Linear Regression (MLR) through extended ANOVA statistical treatment were applied in a similar way as previously reported [24,25]. The main in vitro oil descriptors were included in a PCA analysis. Single value decomposition analysis was applied for PCA and the AUC Δoil, AUC Δhydration and AUC Δelasticity descriptors were analysed by a second PCA. In the MLR studies, the in vitro physicochemical characteristics of the oils were included as predictors for the in vivo performance, selected as responses in this analysis. The Kernels logarithm was applied to establish the correlation between predictors and responses. A *p*-value of < 0.05 was considered as significant.

## 3. Results and Discussion

Table 2 shows the mean results and the standard deviation obtained for the in vitro characterisation of the oils tested in this study. The differences in density, dynamic viscosity, melatonin carrier capacity (expressed as P_lipid/water_ coefficient) and initial peroxide values between the oils were statistically significant (*p* < 0.001). However, the characteristics of WOO were not statistically (*p* > 0.1) different from the two other olive oils (EVOO and VOO).

The degree of unsaturation of the oils is a key chemical parameter. LP does not have any unsaturated acids in its composition; therefore, its initial Ip value is 0 and it is very stable, even when in contact with H_2_O_2_, as demonstrated by the value for oil oxidation under stress ΔIp (%), shown in Table 2. However, unsaturation is also related to the melatonin carrier capacity in the oil phase, and this parameter is very low for LP.

The ratio of unsaturated oils for RO and olive oils is usually higher than 80%. In olive oils, the ratio of oleic acid is approximately 70%, linoleic acid is 10% and palmitoleic acid is about 2%. Jimenez-Lopez et al. [29] have reported an 82% unsaturated acid oil composition for olive oils. In RO, it is even higher, and over 90% of its composition consists of unsaturated acid oils, with linoleic acid as the main component, with 54% [30]. The unsaturation composition of the tested oil can be seen from the initial peroxide (Ip) values, shown in Table 1. The higher the unsaturation composition of the oils (Ip values), the higher the melatonin carrier capacity, expressed as the P_lipid/water_ coefficient. However, a higher unsaturation composition (Ip values) is also related to poor oil stability when in contact with oxidant agents, as indicated by the values for oil oxidation under stress ΔIp (%).

Melatonin is a natural endogenous antioxidant and anti-inflammatory product with poor stability [31]. In contact with H_2_O_2_, its half-life is approximately eight days, and on day 11, more than 50% is degraded without any significant (*p* > 0.1) differences based on the type of oil. However, significant oil-dependent differences are observed after one day of contact between melatonin and the oxidant medium (*p* < 0.01). WOO and RO have a more efficient antioxidant melatonin protection action than LP, suggesting the presence of more powerful antioxidant components.

**Figure 1:**
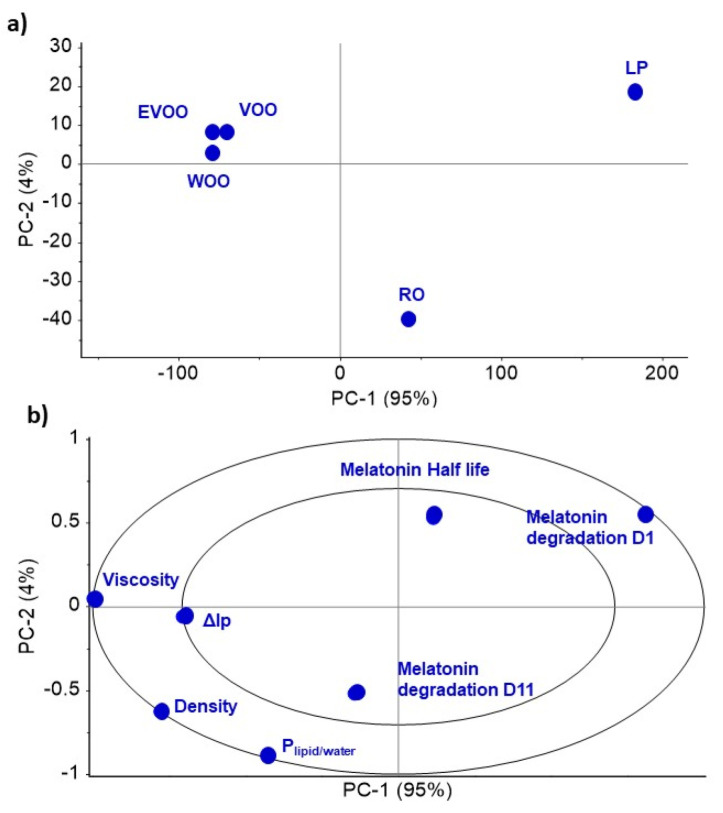
PCA analysis of the in vitro characteristics of the oils. (**a**) Scores; (**b**) Correlation loadings.

The same correlation was observed in the PCA analysis. The three types of olive oils are grouped together and have a similar pattern of in vitro characteristics (Figure 1a). LP shows the greatest difference; it is mainly composed of alkanes with linear and saturated oils. The other oils have a high ratio of unsaturated oils, giving them a higher density and less chemical stability than LP. RO is also an unsaturated oil but behaves differently to olive oils. A clear inverse correlation is observed between the P_lipid/water_ and melatonin degradation on day one (Figure 1b), supporting the evidence that the higher the unsaturation, the higher the P_lipid/water_, which triggers an enhanced antioxidant protection of melatonin and higher oil oxidation under stress (ΔIp). However, no relationship is found with the degradation on day 11 due to the high degradation percentage. 

**Figure 2:**
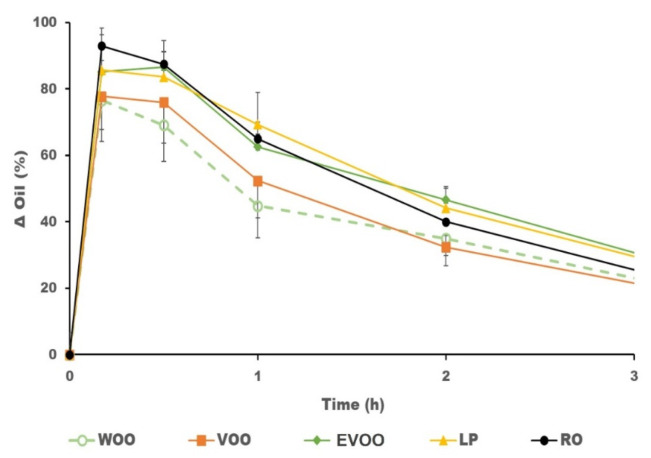
Mean results and variability (*n* = 8) in the increase in oil (%) at the site of administration during the first 3 h after application.

The in vivo evaluation of the effects of the oils in the skin of the volunteers was quantified with a dermoanalyser and the results are summarised in Table 3. Figure 2 shows the oil increase in the skin after the topical administration of the different liquid excipients. WOO produced less increment of oil after application. This result was aligned with the volunteers’ perception, who reported a less oily sensation after the topical application of WOO than with the other oils.

**Figure 3:**
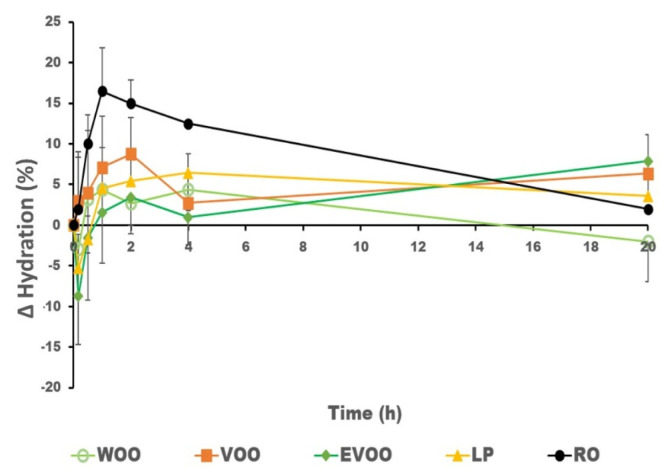
Mean results and variability (*n* = 8) in the increase in hydration (%) at the site of administration during the first 20 h after application.

**Figure 4:**
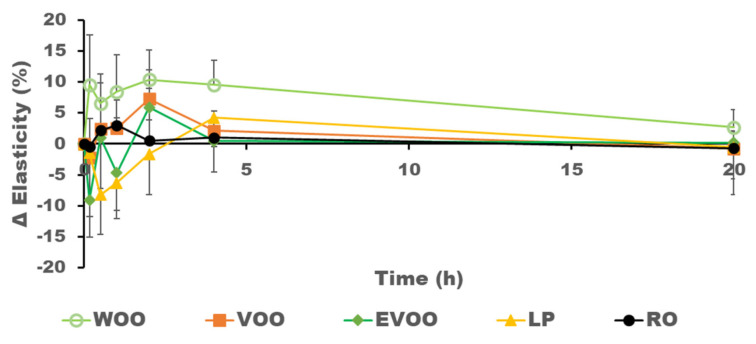
Mean results and variability (*n* = 8) in the increase in elasticity (%) at the site of administration during the first 20 h post-application.

Figure 2 and Figure 3 show the hydration and elasticity after the oil was applied on the volunteers’ skin. The initial negative values of increments of hydration observed in some volunteers after the administration of the oil may be related to a decrease in conductivity due to the presence of the oil on the surface, which interacts with the assessment of the skin’s hydration. There is generally a direct relationship between the presence of oil in the skin and its hydration level. RO shows the highest presence in the skin and the greatest increment in hydration, while WOO has less presence in the skin and the lowest increase in hydration, as can be seen in Figure 4. WOO produces the highest increase in elasticity after topical application.

**Figure 5:**
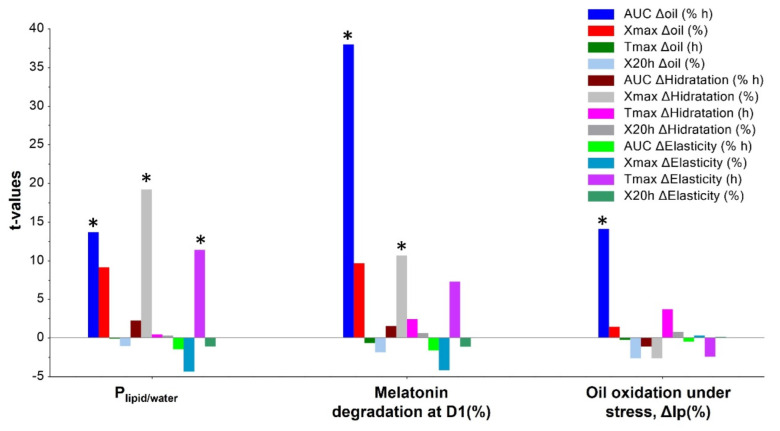
t-values from the MLR analysis of P_lipid/water_, melatonin degradation at day 1 and oil oxidation under stress (%). Key: * *p*-value < 0.05.

Figure 5 shows the t-values from the MRL analysis of P_lipid/water_, melatonin degradation at day one, and oil oxidation under stress. The other in vitro characteristics did not significantly correlate with the responses included in this analysis. A direct correlation was observed between the P_lipid/water_ and the AUC Δoil, Xmax ΔHydration, and tmax ΔElasticity. RO has the highest melatonin carrier capacity, shown in Table 2 as the melatonin P_lipid/water_ coefficient. The RO has a carrier capacity that is two-times more significant than the three types of olive oils, and 30-times larger than LP. RO exhibited the highest values for Xmax ΔHydration and tmax ΔElasticity, meaning it is the oil that produces the highest increment in hydration in the skin and maintains its elasticity for longer. High oil oxidation under stress also correlates significantly with higher oil retention in the skin, which explains the high AUC Δoil for EVOO.

**Figure 6:**
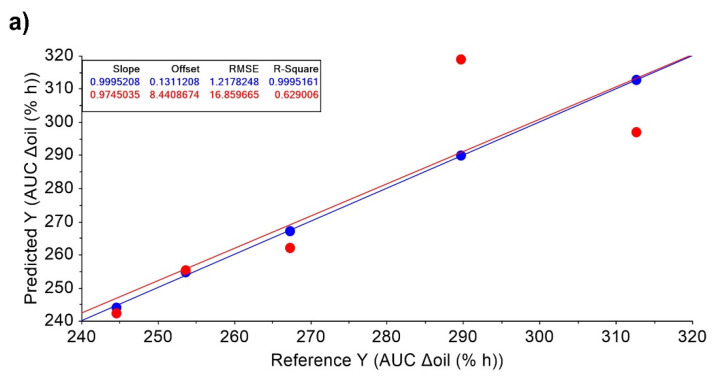

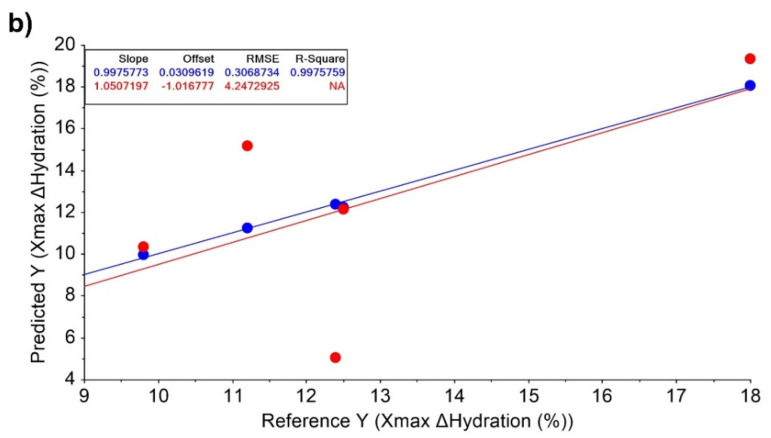
MLR models and fitting parameters for calibration and validation data for: (**a**) AuC Δoil and (**b**) Xmax ΔHydration. Blue and red circles represent calibration and validation data, respectively for MRL models. RMSE: Root Mean Square Error.

Interestingly, a high melatonin degradation on day one relates to the high retention of oil in the skin and an increase in hydration. These features are associated with LP, which has a lower antioxidant capacity than the other oils, resulting in less protection against melatonin degradation. However, as an occlusive emollient it creates a water-resistant barrier on the skin that minimises water loss and retains hydration for longer [32]. A direct correlation with a high determination coefficient (R^2^~1) was observed for AUC Δoil and Xmax Δhydration (Figure 6).

**Figure 7:**
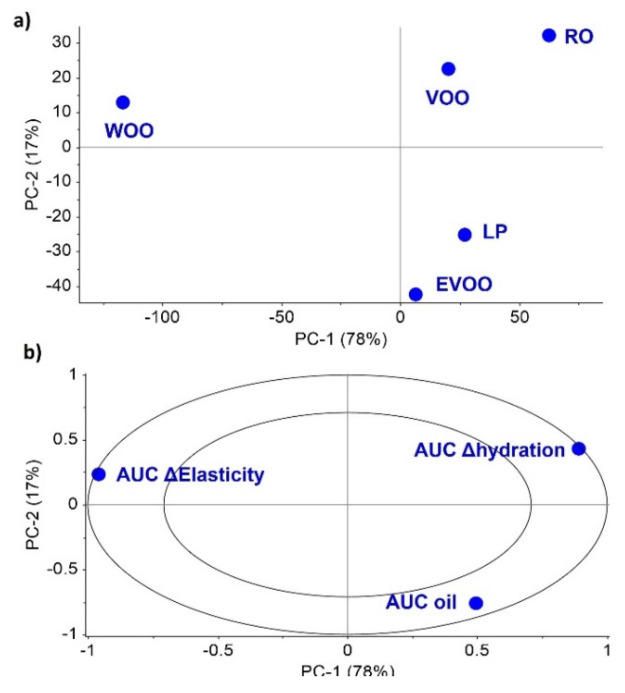
PCA analysis of AUC Δoil, AUC Δhydration and AUC Δelasticity features. (**a**) Scores; (**b**) Correlation loadings.

LP has the lowest density and viscosity, and the highest increase in oil at the site of application (see AUC Δoil results shown in Table 3). The dermoanalyser provides results at different times, and the AUC values are considered the most relevant because they include individual data from the application for up to 20 h. The results of both AUC Δoil and AUC Δhydration are relatively close, suggesting a direct relationship. It is well known that oil in the skin is related to its hydration. However, the parameter AUC Δelasticity is independent of the other two AUCs parameters (AUC Δoil and AUC Δhydration). AUC Δelasticity has an inverse correlation to the other two AUCs parameters in the space of the correlation loadings, as shown in Figure 7. Interestingly, the PCA for the AUC Δoil, AUC Δhydration, and AUC Δelasticity indicates that WOO behaves very differently compared to the other two olive oils. This may explain the results obtained from the in vivo analysis, in which WOO shows the poorest AUC Δoil and AUC Δhydration values but the highest AUC Δelasticity value.

It is well known that skin hydration in the stratum corneum plays a crucial role in skin condition and, vice versa, that its hydration level influences the skin’s efficacy properties. However, few studies have been performed to establish a correlation between modifications in skin hydration content and skin characteristics at different skin depth [33]. For example, the skin hydration measured by the dermoanalyser penetrates to a depth of 10–20 µm of the stratum corneum [28]. It has been demonstrated that skin elasticity is influenced by its hydration level at viable epidermis depths, while skin roughness is more affected by hydration at the stratum corneum level [33]. This highlights the importance of determining the effect of topically applied formulations on hydration at the stratum corneum and of monitoring skin elasticity or the depth profile of water content using other techniques such as Raman spectroscopy. Based on the results obtained in this study, compared to other olive oils, WOO appears to be highly effective in reaching deeper areas of the skin and enhancing skin elasticity. This characteristic may be related to its excellent permeability across the stratum corneum, which explains the lower levels of oil on the skin surface and its lower skin hydration than the other olive oils in the study. As skin elasticity is considered the critical indicator of skin ageing [34], WOO is therefore a promising alternative for formulating novel topical cosmetic anti-aging formulations.

## 4. Conclusions

The in vitro results of the oils are closely related to their degree of unsaturation. However, as potential carriers for molecules with a high tendency of oxidation, RO and WOO can be considered suitable excipients because of their antioxidant protective effects.

The in vivo results generally showed a direct relationship between the effects of the oil and hydration. However, an inverse relationship between oil and hydration is observed for elasticity. LP shows the highest oil increment in the skin, creating an occlusive barrier that prevents water loss from the skin, while WOO has the lowest increment of oil in the skin but the highest increase in elasticity, indicating deeper skin penetration and better skin performance overall.

## Figures and Tables

**Table 1:** Cross-over design. Key: Wild Olive Oil (WOO), Extra Virgin Olive Oil (EVOO), Virgin Olive Oil (VOO), Liquid Paraffin (LP) and Rosehip Oil (RO).

Subject	Period 1	Period 2	Period 3	Period 4	Period 5
1	LP	RO	WOO	EVOO	VOO
2	LP	RO	WOO	EVOO	VOO
3	VOO	LP	RO	WOO	EVOO
4	VOO	LP	RO	WOO	EVOO
5	WOO	EVOO	LP	RO	VOO
6	WOO	EVOO	LP	RO	VOO
7	RO	WOO	VOO	LP	EVOO
8	RO	WOO	VOO	LP	EVOO

**Table 2:** Mean results ± standard deviation (*n* = 3) of the in vitro characterisation of the tested oils.

Parameter	RO	LP	EVOO	WOO	VOO
Density (mg/mL)	930.3 ± 1.1	844.5 ± 2.3	912.8 ± 3.4	917.3 ± 2.3	913.4 ± 2.8
Dynamic Viscosity (mPa s)	279.1 ± 31.2	157.6 ± 3.8	408.9 ± 10	410.3 ± 31.4	404.9 ± 14.5
Melatonin P_lipid/water_ coefficient	0.31 ± 0.02	0.01 ± 0.002	0.17 ± 0.01	0.14 ± 0.01	0.17 ± 0.002
Melatonin degradation day 1 (%)	3.5 ± 1.9	22.8 ± 0.2	8.6 ± 2.6	3.2 ± 0.9	4.9 ± 1.9
Melatonin degradation day 11 (%)	65.5 ± 3	59.4 ± 2.6	65.6 ± 5.6	63.5 ± 1.7	55.8 ± 0.3
Melatonin degradation half-life (days)	7.2	9.1	7.1	8.1	10.2
Initial Peroxide Value Ip (mEq O/kg)	40 ± 2.1	0.9 ± 0.3	23 ± 3.6	20.2 ± 0.6	8.1 ± 0.7
Oil oxidation under stress ΔIp (%)	16 ± 4.2	0.0 ± 1.5	42.5 ± 2.8	17.5 ± 4.5	14.1 ± 2.3

**Table 3:** In vivo performance of the various oils tested in eight healthy volunteers. Mean results ± standard deviation.

Parameter	RO	LP	EVOO	WOO	VOO
AUC Δoil (% h)	267.3 ± 90.4	312.7 ± 140.9	289.7 ± 51.6	244.6 ± 144.2	253.7 ± 128.3
Xmax Δoil (%)	94 ± 30.4	90.7 ± 20.9	89.4 ± 40	79.9 ± 23.1	83 ± 27.4
Tmax Δoil (h)	0.33 ± 0.23	0.22 ± 0.12	0.3 ± 0.18	0.31 ± 0.18	0.47 ± 0.34
X20h Δoil (%)	0 ± 0.1	0.4 ± 0.2	−2.2 ± 1.8	1.1 ± 0.7	2.2 ± 1.3
AUC ΔHydration (% h)	168 ± 28.9	116.2 ± 59.9	75.3 ± 59.4	41.7 ± 26.4	128.3 ± 75.4
Xmax ΔHydration (%)	18 ± 8.5	11.2 ± 6.1	12.4 ± 5	9.8 ± 7.3	12.5 ± 9.4
Tmax ΔHydration (h)	2.5 ± 2.11	5.33 ± 4.31	9.31 ± 7.08	1.35 ± 0.91	3.46 ± 2.71
X20h ΔHydration (%)	2 ± 0.2	3.6 ± 2.1	7.9 ± 4.1	−2 ± 0.9	6.4 ± 4.2
AUC ΔElasticity (% h)	7.2 ± 5.8	22.9 ± 11.4	9.2 ± 7.2	133.8 ± 90.1	26.3 ± 19.1
Xmax ΔElasticity (%)	7.8 ± 6.3	10.4 ± 8.5	12.6 ± 7	19.1 ± 13.4	13.2 ± 9.9
Tmax ΔElasticity (h)	11 ± 9.72	4.48 ± 4.03	4.14 ± 3.1	1.78 ± 1.41	3.8 ± 2.66
X20h ΔElasticity (%)	−0.7 ± 0.6	−0.53 ± 0.4	0.19 ± 0.15	2.7 ± 2.1	−0.7 ± 0.4

## Data Availability

Data is contained within the article.
